# Palmitic acid supplementation enhances milk fat synthesis and energy balance without altering intake or yield in lactating goats

**DOI:** 10.14202/vetworld.2025.3670-3683

**Published:** 2025-12-07

**Authors:** Jenny Nathalia Álvarez-Torres, Jacinto Efrén Ramírez-Bribiesca, Yuridia Bautista-Martínez, Alexis Ruiz-González, María Magdalena Crosby-Galván, Mónica Ramírez-Mella, Jorge Alonso Maldonado-Jáquez, Lorenzo Danilo Granados-Rivera

**Affiliations:** 1 Programa de Ganadería, Colegio de Postgraduados Campus Montecillo, Texcoco, Texcoco de Mora, 56230, Mexico; 2 Facultad de Medicina Veterinaria y Zootecnia, Universidad Autónoma de Tamaulipas, Km. 5 Carretera a Mante s/n, Ciudad Victoria, Tamaulipas, 87000, México; 3 Département des Sols et de Génie Agroalimentaire, Université Laval, Québec City, QC G1V 0A6, Canada; 4 Department of Animal Production, National Technological Institute of Mexico, Technological Institute of Chiná. Chiná, Campeche, 24520, Mexico; 5 Department of Animal Genetics, National Institute of Forestry, Agricultural and Livestock Research. La Laguna Experimental Field. Matamoros, Coahuila, 27440, Mexico; 6 Department of Animal Nutrition, National Institute of Forestry, Agricultural, and Livestock Research. General Terán Experimental Field. General Terán, Nuevo León, 67400, Mexico.

**Keywords:** dairy goats, energy balance, fatty acid profile, milk composition, palmitic acid, rumen-inert fat

## Abstract

**Background and Aim:**

Palmitic acid (PA) (C16: 0) is a rumen-inert long-chain fatty acid (FA) widely used in dairy cattle to increase dietary energy density and milk fat synthesis; however, its effects in dairy goats remain poorly characterized. This study evaluated whether supplementing 3% or 6% PA in the diet of mid-lactation goats could improve milk yield, composition, FA profile, and whole-animal energy balance under semi-arid Mexican production conditions.

**Materials and Methods:**

Twenty-one multiparous crossbred goats (45.8 ± 1.2 kg; 21 ± 3 days in milk) were randomly assigned to three treatments for 6 weeks after a 2-week adaptation: (1) Control diet (without PA), (2) diet + 3% PA, and (3) diet + 6% PA on a dry-matter (DM) basis. Diets were isoenergetic and isoproteic before PA addition. Individual DM intake (DMI), milk yield, and composition were measured daily; milk FA profiles and energy balance were determined on days 0, 21, and 42. Data were analyzed using a mixed-model with repeated measures, and means were compared using the Tukey test (p ≤ 0.05).

**Results:**

PA inclusion did not affect DMI, body weight, or milk yield. However, milk fat concentration and yield increased significantly (p < 0.01) in both PA treatments, with the highest fat concentration observed at 6% PA. The milk FA profile shifted toward greater C16: 0 and C16: 1 proportion (p < 0.0001) and decreased short-chain (<C16) and long-chain (>C16) FA fractions. Energy-corrected milk yield rose by ~40% in PA-fed goats, and energy balance improved markedly from week 3 onward, particularly in the 3% group (p < 0.01), indicating superior dietary energy utilization without intake suppression.

**Conclusion:**

Moderate PA supplementation (~3% DM) effectively enhances milk fat synthesis and energy efficiency in goats while maintaining stable intake and yield. Increasing PA beyond 3% confers minimal additional benefit and may overly saturate milk fat. These findings provide species-specific evidence that rumen-inert fat inclusion can be an efficient strategy to support metabolic status and product quality in mid-lactation goats under variable forage systems.

## INTRODUCTION

Goats are a versatile livestock species that make a substantial contribution to global agricultural sustainability and food security [1]. They are particularly well adapted to arid, semi-arid, and mountainous environments where other livestock species struggle to survive [2]. In Mexico, goats represent an integral component of smallholder farming systems, providing meat, milk, and hide that support rural livelihoods and household nutrition [3].

Goat milk, in particular, has attracted increasing attention for its superior nutritional profile and therapeutic potential in addressing malnutrition and specific health disorders. It is characterized by high digestibility, low allergenicity, and a rich supply of essential nutrients, including high-quality proteins, vitamins, and minerals [1]. Furthermore, it contains bioactive lipids such as conjugated linoleic acid and monounsaturated fatty acids (FAs) [4], which are associated with beneficial effects on cardiovascular health and lipid metabolism [5]. Its reduced αS1-casein content also makes goat milk less allergenic than cow milk [6], offering a suitable alternative for individuals with milk protein sensitivities or lactose intolerance [7].

In dairy ruminants, the onset of lactation poses a significant metabolic challenge because energy intake through dry matter intake (DMI) often fails to meet the heightened demands for maintenance (net energy for maintenance, NEM) and milk synthesis (net energy for lactation [NEL]). This imbalance results in a negative energy balance (NEB), compelling animals to mobilize body reserves to sustain milk production, with potential consequences for health and productivity [8]. To alleviate NEB, nutritional strategies to increase dietary energy density have been extensively studied, particularly in dairy cattle [9].

Palmitic acid (PA) (C16: 0) is the predominant FA in goat milk and a key substrate for milk fat synthesis [9]. Among long-chain FAs (LCFAs), PA demonstrates the highest transfer efficiency from diet to milk fat in ruminants [10]. In dairy cows, dietary supplementation with rumen-inert PA has been shown to enhance milk yield, increase milk fat concentration, and improve overall energy balance [11, 12]. However, despite this robust evidence in cattle [10–13], corresponding research in dairy goats remains scarce [14]. Goats differ markedly from cows in mammary lipid metabolism; they produce more *de novo* short-and medium-chain FAs (C6: 0–C12: 0), exhibit distinct regulatory sensitivity of mammary lipogenesis to preformed LCFA, and display a lower incidence of diet-induced milk fat depression [1–8]. Consequently, responses observed in bovine models cannot be extrapolated directly to caprine systems [4].

In northern and central Mexico, dairy goat herds are predominantly composed of local crossbreds managed under fluctuating forage quality and semi-arid climatic conditions, factors that critically influence nutrient partitioning, lipid metabolism, and feeding efficiency [3]. Evaluating PA supplementation in such herds provides context-specific insights into feed intake, milk fat composition, and energy balance under practical field conditions, which are not adequately represented by confined Holstein cow studies.

Despite the well-documented role of PA in improving milk fat synthesis and energy balance in dairy cows, there remains a significant knowledge gap regarding its biological efficacy, optimal inclusion level, and metabolic impact in dairy goats. Most existing studies have focused on bovine models under intensive feeding systems, yet the metabolic regulation, lipid partitioning, and mammary responses of goats differ markedly from those of cattle. This divergence in lipid metabolism challenges the direct extrapolation of bovine results to caprine production systems.

Previous research on goats has been limited in scope, often using single supplementation levels, short experimental periods, or small sample sizes, which preclude robust conclusions. Furthermore, many trials have failed to evaluate energy balance and milk FA partitioning simultaneously, thereby overlooking the link between dietary lipid inclusion, mammary substrate use, and systemic energy dynamics.

Another unaddressed aspect is the interaction between PA supplementation and variable feeding conditions typical of semi-arid and smallholder production systems in regions such as northern Mexico. Goats in these systems are commonly fed mixed or forage-based diets with fluctuating nutrient quality, which creates distinct metabolic and physiological pressures compared with confined, high-input dairy systems. Consequently, the real-world nutritional value and metabolic efficiency of PA supplementation in such environments remain uncertain.

Moreover, no prior studies have comprehensively assessed the graded response of 0%, 3%, and 6% dietary PA inclusion in goats, especially with respect to milk FA profile modulation, energy-corrected milk (ECM) yield, and overall energy balance. The absence of such comparative, multi-level assessments has limited the ability to identify the threshold at which PA shifts from beneficial to biologically redundant or economically inefficient.

Therefore, a controlled investigation focusing on graded PA supplementation under realistic production conditions is essential to clarify:

- The dose-dependent effects of PA on milk composition and energy utilization.
- The species-specific metabolic handling of LCFAs in goats.
- The practical feeding recommendations applicable to variable forage systems in semi-arid regions.

Addressing these gaps provides not only mechanistic insights into caprine lipid metabolism but also evidence-based guidance for optimizing dietary fat use in goat production systems where energy density, rather than maximum yield, is the primary constraint.

This study, therefore, aimed to assess the graded inclusion of PA (0%, 3%, and 6% of dietary dry matter [DM]) in mid-lactation goats. Given the unique features of caprine mammary lipid metabolism, (1) strong *de novo* synthesis control through acetate/β-hydroxybutyrate (β-hydroxybutyrate) pathways and ACC/FASN feedback regulation, (2) distinct partitioning and esterification favoring C16: 0 incorporation without yield escalation, and (3) higher tolerance to moderate dietary fat without classical milk fat depression, the study was designed to determine whether moderate PA inclusion could optimize the milk FA profile and enhance energy balance without compromising intake. By focusing on local Mexican crossbred goats under controlled feeding and milking conditions, this work provides species-and context-specific evidence addressing a critical gap left by cattle-focused nutritional research.

## MATERIALS AND METHODS

### Ethical approval

All experimental procedures were conducted in accordance with institutional and national regulations for the care and use of animals in research. The protocol was approved by the Institutional Committee for the Care and Use of Animals in Research, Colegio de Postgraduados (approval no. COBIAN/009/23).

Goats were individually housed in pens (2 × 3 m) equipped with shaded areas, individual feeders, and free access to clean water. Animals were inspected twice daily (a.m. /p.m.) for general health, rumen fill, hydration, and locomotion. Any deviations triggered immediate veterinary examination, and animals were temporarily removed from data collection until recovery. Handling procedures, including weighing, feeding adjustments, and sampling, were performed by trained staff using low-stress techniques (calm approach, minimal restraint, and no electric prods). Body weight was recorded weekly with a calibrated portable scale (capacity 200 kg; ±10 g, Model FS200-AI, Torrey, Mexico). To minimize stress, all procedures were conducted by the same technicians at consistent times.

### Study period and location

The study was conducted from September to November 2024 at a private livestock unit dedicated to goat breeding. This unit is located in the Zaragoza ejido in the municipality of Viesca in the Mexican state of Coahuila.

### Experimental site and climate

The study was conducted in a private goat unit located in the Zaragoza Ejido, municipality of Viesca, Coahuila, Mexico (1100 m above sea level). The regional climate is classified as warm and dry (BWh), with an average annual temperature of 22.1°C and an average annual precipitation of 261.4 mm. During the experimental period, the average ambient temperature and relative humidity were 23°C and 52.5%, respectively.

### Animals and experimental design

Twenty-one crossbred local goats (third lactation; 21 ± 3.4 days in milk) with an average body weight of 45.82 ± 1.19 kg were used during early lactation. All animals were clinically healthy and had undergone a preventive health program including deworming and vaccination against pneumonic mannheimiosis, helminthiasis, and diarrhea.

Goats were randomly assigned to three groups (n = 7) in a completely randomized design. The experimental period included a 2-week adaptation phase followed by 6 weeks of data collection. Treatments were as follows:

- NPA (control): Basal diet formulated to meet National Research Council (NRC) [15] nutrient requirements (Table 1).
- PA3: Basal diet + 3% PA (Jefo Dairy Fat 99%) on a DM basis.
- PA6: Basal diet + 6% PA on a DM basis.

**Table 1:** Chemical composition and FA profile of the components of the basal diet (mean ± SEM).

Chemical composition	(% DM)	Supplement^b^
DM	94.6 ± 4.6	-
Crude protein	12.2 ± 1.2	-
Neutral detergent fiber	49.5 ± 7.1	-
Acid detergent fiber	30.2 ± 4.2	-
ME^a^ (Mcal/kg DM)	1.8 ± 0.3	12.8 ± 1.1

**FAs (Total FA or g/100 g DM)**	**(% Basal diet)**	**Supplement**

<C16:0	2.55 ± 0.3	2.75 ± 0.2
C16:0	24.54 ± 3.4	86.53 ± 14.3
C16:1	1.50 ± 0.2	-
C18:0	2.94 ± 0.7	3.57 ± 0.1
C18:1 n-9	16.68 ± 4.2	6.17 ± 1.1
C18:2 n-6	18.74 ± 3.9	0.98 ± 0.1
C18:3 n-3	32.28 ± 9.7	-
C20:0	0.77 ± 0.1	-

a = Calculated according to the National Research Council [15], b Supplement = Jefo dairy fat 99%, SEM = Standard error of mean, DM = Dry-matter, FA = Fatty acid, ME = Metabolizable energy.

Diets were formulated to be isoenergetic and isoproteic before fat inclusion.

### Diet and feeding management

Rations were offered individually, and feed refusals were collected and weighed daily before the morning feeding to calculate DM intake. Feed allowances were adjusted weekly according to each goat’s live weight to maintain consistent intake levels.

### Housing and milking procedures

Goats were milked once daily at 09:00 a.m. in a consistent order throughout the trial. During milking and sample collection, animals were briefly restrained in a gate to ensure operator safety and sample integrity. Restraint time was minimized, and good milking hygiene practices were strictly followed to ensure milk quality.

### Measurements and data collection

#### Milk sampling and processing

Daily milk yield was recorded at each milking. Milk was thoroughly mixed before sampling, and duplicate aliquots (2 × 50 mL) were collected into sterile polypropylene tubes (Falcon, USA), one for immediate analysis and the other stored for confirmation.

For FA analysis, 50 mL of milk was placed in amber polypropylene tubes (Eppendorf Tubes, USA), kept on ice, and transported to the laboratory within 1 h. Samples were stored at −20°C until batch processing for the preparation of FA methyl esters (FAMEs). Each batch included blanks and certified standards (Nu-Check Prep, 37-component mix). Internal quality-control samples (pooled milk) were analyzed every 10 samples to ensure analytical consistency (±2 standard deviation of the historical mean). FA results were expressed as g/100 g of total FA.

All sample tubes were labeled with animal ID, date, time, and sample type. Chain-of-custody and cold-chain integrity were maintained from field collection through analysis.

### Body weight and DM intake

Body weight was recorded weekly before morning feeding, using calibrated scales (Model FS200-AI. Torrey). Daily dry matter intake (DMI) was calculated as the difference between DM offered and DM refused.

### Milk production and composition

Milk yield and composition (fat, protein, and lactose) were determined individually using a portable digital scale (capacity 10 kg ± 1 g, Torrey EQB, Mexico) and a Milkoscan analyzer (Lacticheck Model LC-01, USA). Measurements were conducted throughout the experimental period.

### Sample collection and laboratory analyses

#### Milk composition

Milk protein, fat, and lactose were quantified using infrared spectrophotometry (Lacticheck Model LC-01) at the INIFAP Dairy Laboratory, Matamoros, Coahuila.

#### Feed composition

The chemical composition of the basal diet and the FA profile of feed ingredients and supplements were determined at the Animal Nutrition Laboratory, Colegio de Postgraduados (Montecillo Campus). Standard methods were used for DM (#930.15), crude protein (#984.13), ether extract (#920.29) [16], neutral detergent fiber, and acid detergent fiber [17].

#### FA profiling

Milk and feed FA profiles were determined by Feng *et al*. [18] and the modified methylation method by Granados-Rivera *et al*. [19]. For feed, 0.5 g samples were analyzed; for milk, 50 µL lipid extracts were used. Samples were treated with sodium methoxide (0.5 M in methanol), vortexed, heated (80°C for 10 min), cooled, and extracted with hexane and potassium carbonate. After drying and filtration (0.45 µm nylon membrane), FAMEs were analyzed using a Hewlett-Packard 6890 GC (Hewlett-Packard 6890, USA) equipped with a Sp-2560 column (100 m × 0.25 mm × 0.20 µm). Oven temperature was programmed from 100°C to 235°C (5°C/min). Helium served as the carrier gas (32 cm/s). FAs were identified by retention times relative to the standard mix (Nu-Check Prep, Nu-Check, USA).

### Energy balance calculation

ECM yield was calculated as described by Rico *et al*. [12]:

ECM (kg/day) = [0.327 × milk yield (kg/day)] + [12.95 × fat (kg/day)] + [7.65 × protein (kg/day)].

Energy balance (Mcal/day) was assessed on weeks 1, 3, and 6 using NRC guidelines [20]:

Energy balance = ME intake − (MEM + MEL),

Where ME = Metabolizable energy, MEM = Maintenance requirement, MEL = Lactation requirement.

ME was computed from total digestible nutrients (TDN) as described by Weiss *et al*. [21]:

ME (Mcal/kg) = TDN × 4.4 × 0.82.

MEM was calculated as 110 kcal × BW0.75 [22], and MEL was calculated using:

MEL (Mcal/day) = (milk production × [0.3512 + 0.0962 × milk fat% %])/0.589 [23].

### Statistical analysis

All analyses were performed using SAS v.8 (SAS Institute, USA). Data were analyzed using a completely randomized design with repeated measures using the PROC MIXED (SAS Institute, USA) procedure. FA data (weeks 1, 3, and 6) and other continuous variables were modeled separately.

Multicollinearity among fixed effects (treatment, week, and interaction) was verified using variance inflation factors (VIF < 5). Model assumptions were checked through Shapiro–Wilk normality tests, Q–Q plots, and residual diagnostics. Homoscedasticity was assessed by Levene and Brown–Forsythe tests. Candidate covariance structures (CS, AR [1], UN) were compared through AIC values, and the best-fitting structure was selected. Influential data points were evaluated using studentized residuals and Cook’s distance (|r|> 3 or D > 4/n).

If assumptions were violated, corrections (heteroscedasticity-consistent residual variances or log transformations) were applied and justified. Treatment means were compared using Tukey’s test, and statistical significance was declared at p ≤ 0.05.

## RESULTS

### Body weight, feed intake, and milk production

Table 2 summarizes the body weight, dry matter intake (DMI), and milk production performance of goats under different dietary treatments. No significant differences (p > 0.05) were observed among groups for body weight, DMI, or milk yield. However, goats supplemented with PA produced significantly higher ECM (p < 0.05) than those in the control (NPA) group.

**Table 2:** Average body weight, DMI, milk production, and composition in goats treated with different doses of palmitic acid.

Variable	Treatment			SEM	p-value	
	
	NPA	PA3	PA6		PA	PA × week
Body weight (kg)	46.48	45.69	46.64	2.91	0.97	0.33
DM consumed (kg/day)	2.22	2.24	2.08	0.15	0.71	0.69
Milk production (g/day)	1453.89	1788.28	1579.17	124.17	0.19	0.63
ECM (g/day)	1332.03^b^	1884.5^a^	1848.64^a^	158.47	0.04	0.53
Milk composition (%)						
Protein	3.31	3.35	3.28	0.12	0.92	0.15
Fat	2.63^b^	3.61^ab^	4.51^a^	0.36	0.007	0.94
Lactose	4.91	5.02	4.8	0.18	0.7	0.01
Yield (g/day)						
Protein	48.18	60.56	53.99	5.24	0.28	0.03
Fat	37.68^b^	64.59^a^	69.3^a^	6.66	0.009	0.98
Lactose	71.55	90.47	80.46	7.79	0.26	0.01

^a,b,c^Means with different letters in the column indicate differences (Tukey p ≤ 0.05), SEM = Standard error of the mean, PA = Palmitic acid, NPA = Basal diet treatment, PA3 = Basal diet + 3% inclusion of PA according to the DM offered, PA6 = 6% inclusion of PA according to the DM offered, ECM = Energy corrected milk, DMI = Dry matter intake, DM = Dry-matter.

### Milk fat concentration and yield

Fat concentration and yield were strongly influenced by PA inclusion levels. The highest fat concentration was recorded in goats receiving the 6% PA diet (PA6), while both PA3 and PA6 treatments achieved significantly greater (p < 0.05) fat yields than the control. Fat yield was comparable between the PA3 and PA6 groups, indicating that increasing PA beyond 3% offered limited additional benefit in terms of fat output.

### Milk lactose and protein dynamics

**Figure 1:**
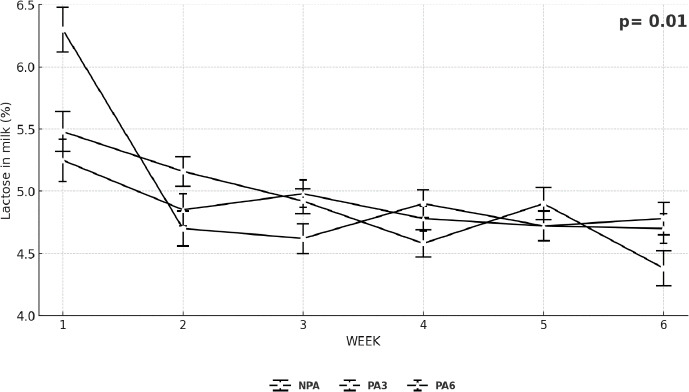
Weekly variation in lactose concentration in lactating goats fed a basal diet (Without PA) or supplemented with two doses of PA (3% and 6% of DM intake). p ≤ 0.05 indicates treatment × week interaction. PA = Palmitic acid, DM = Dry matter.

**Figure 2:**
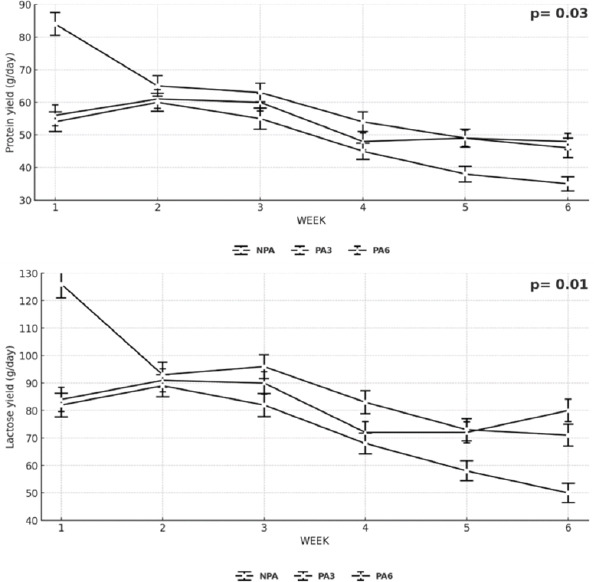
Weekly variation in protein and lactose yield of lactating goats fed a basal diet (Without PA) or supplemented with two doses of PA (3% and 6% of DM intake) p ≤ 0.05 indicates treatment × week interaction. DM = Dry-matter, PA = Palmitic acid.

The lactose concentration (Figure 1) decreased from week 2 onward in goats supplemented with 3% PA. Similarly, lactose yield (Figure 2) declined progressively from week 3 to 6 across treatments, except for PA6, where a slight recovery was observed beginning in week 4.

Protein yield (Figure 2) in PA6 goats initially decreased up to week 3 but gradually increased between weeks 4 and 6, suggesting a partial adaptive response over time. Overall, variations in protein and lactose reflected subtle metabolic adjustments in response to dietary lipid inclusion.

### Milk FA composition

The milk FA profile (Table 3) was markedly altered by PA supplementation. The proportions of *de novo* synthesized FAs (<C16) and preformed LCFAs (>C16) decreased as dietary PA levels increased. In contrast, the concentrations of C16: 0 (PA) and C16: 1 rose significantly with both PA inclusion rates, confirming an efficient dietary transfer of PA to milk fat and a shift toward medium-chain lipid predominance.

**Table 3:** FA profile (g/100 g of total FA) of milk from lactating goats supplemented with two doses of palmitic acid.

FA	Treatments			SEM	p-value	
	
	NPA	PA3	PA6		PA	PA × week
C6:0	3.03	2.90	2.80	0.14	0.52	0.03
C8:0	3.73^a^	3.09^b^	2.55^c^	0.14	<0.0001	0.07
C10:0	14.48^a^	10.69^b^	8.89^c^	0.48	<0.0001	0.66
C11:0	0.36^a^	0.29^ab^	0.24^b^	0.03	0.02	0.41
C12:0	6.96^a^	4.70^b^	3.91^b^	0.25	<0.0001	0.81
C13:0	0.19^a^	0.11^b^	0.09^b^	0.02	0.00	0.57
C14:0	13.42^a^	10.52^b^	9.55^b^	0.38	<0.0001	0.15
C14:1	0.09	0.10	0.09	0.02	0.93	0.42
C15:0	1.08^a^	0.76^b^	0.61^c^	0.04	<0.0001	0.40
C16:0	30.19^c^	41.43^b^	49.29^a^	0.52	<0.0001	0.13
C16:1	0.86^c^	1.25^b^	1.60^a^	0.09	0.00	0.00
C17:0	0.78^a^	0.55^b^	0.38^c^	0.02	<0.0001	0.02
C17:1	0.21	0.19	0.12	0.03	0.06	0.20
C18:0	6.74^a^	6.07^a^	4.71^b^	0.29	0.00	0.03
C18:1 t9	0.89^a^	0.68^b^	0.55^c^	0.04	0.00	0.89
C18:1 c9	14.00	14.07	12.51	0.91	0.41	0.93
C18:2	1.61^a^	1.46^a^	1.17^b^	0.08	0.00	0.61
C18:2 c9t11	0.20^a^	0.15^ab^	0.11^b^	0.01	0.00	0.27
C18:3	0.29^a^	0.25^ab^	0.17^b^	0.03	0.01	0.58
C20:0	0.11^a^	0.10^a^	0.07^b^	0.01	0.00	0.00
C20:4	0.08^a^	0.06^ab^	0.04^b^	0.01	0.00	0.76
Not identified	0.71^a^	0.63^ab^	0.57^b^	0.03	0.02	0.87
<C16	43.33^a^	33.16^b^	28.73^c^	1.21	<0.0001	0.67
C16:0 + C16:1	31.05^c^	42.68^b^	50.89^a^	0.54	<0.0001	0.09
>C16	24.91^a^	23.56^a^	19.83^b^	1.07	0.01	0.74

^a,b,c^Means with different letters in the column indicate differences (Tukey p ≤ 0.05), SEM = Standard error of the mean, PA = Palmitic acid, NPA = Basal diet treatment, PA3 = Basal diet + 3% inclusion of PA according to the DM offered, PA6 = 6% inclusion of PA according to the DM offered, DM = Dry-matter, FA = Fatty acid.

### Energy balance

At the beginning of the experiment, all goats exhibited a slightly NEB (Table 4). From week 3 onward, energy balance improved across all treatments, with PA-supplemented goats demonstrating a stronger recovery trend. The improvement was most evident in goats receiving 3% PA, indicating enhanced energy utilization efficiency relative to the control group.

**Table 4:** Energy balance of goats supplemented with palmitic acid at two levels.

Energy balance (Mcal/day)	Treatment			SEM	p-value

	NPA	PA3	PA6		
Week 1 (day 0)	−0.13	−0.08	−0.11	0.82	0.76
Week 3 (day 21)	0.09^b^	1.51^a^	1.94^a^	0.08	<0.01
Week 6 (day 42)	0.54^b^	1.47^a^	1.78^a^	0.03	<0.01

^a,b,c^ Means with different letters in the column indicate differences (Tukey p ≤ 0.05), SEM = Standard error of the mean, PA = Palmitic acid, NPA = Basal diet treatment, PA3 = Basal diet + 3% inclusion of PA according to the DM offered, PA6 = 6% inclusion of PA according to the DM offered, DM = Dry-matter.

## DISCUSSION

### Intake and milk yield remain stable

The body weight, DMI, and milk production of lactating goats supplemented with PA showed no significant differences across treatments. These findings align with those reported by Lévesque *et al*. [24] and Delavaud *et al*. [25], who observed no effect on milk production in PA-fed goats. However, Piantoni *et al*. [26] reported increased milk production with PA supplementation in dairy cows, which may be attributed to species-specific differences in metabolism [27].

Lévesque *et al*. [24] observed comparable ECM production during mid-lactation in Alpine goats supplemented with 2% PA and those on a control diet. These findings differ from those of the present study, where ECM increased with higher PA inclusion levels. This discrepancy may stem from differences in genetic or phenotypic merit used in ECM estimations and the impact of dietary fat supplementation on milk production efficiency. As noted by Lock *et al*. [28], fat supplementation in ruminant diets can enhance milk production efficiency compared to fat-free diets, with outcomes influenced by the level of fat inclusion.

In the present study, PA supplementation significantly increased both the concentration and yield of milk fat. Similar results have been reported in goats by Lévesque *et al*. [24] and in dairy cows by Piantoni *et al*. [26] and Rico *et al*. [12]. However, Delavaud *et al*. [25] did not observe any significant effects on milk fat when evaluating supplementation with hydrogenated PA at 3% of DM intake in dairy goats.

### Mechanistically shifts milk fat concentration and FA profile

PA appears to promote milk fat synthesis in goat metabolism. This is linked to its ability to increase ceramide concentration, which contributes to insulin resistance and stimulates the release of LCFAs from adipose tissue. These LCFAs are utilized in the synthesis of milk fat [29]. This finding aligns with the work of Palmquist and Jenkins [30], who observed that more unsaturated fats reduce milk fat concentration due to the milk fat depression index. Conversely, the present study demonstrates that dietary PA contributes to milk fat synthesis, particularly by increasing the transfer of C16: 0 FA into milk.

Including PA in the diet effectively stimulated milk fat synthesis, likely due to the efficient transfer of dietary PA to milk fat, with up to 50% of PA being directly transferred [10]. The increase in C16: 0 FA concentration in milk observed in this study suggests a positive transfer of dietary PA, directly contributing to higher milk fat content.

The elongation of FA chains beyond 16 carbons is limited in the mammary gland due to the absence of necessary enzymes [31]. C16: 0 and C18: 0 FAs in milk fat are derived from two main sources: (a) Dietary triglycerides: These are transported through chylomicrons and very low-density lipoproteins and hydrolyzed by lipoprotein lipase, releasing FAs and glycerol, which are absorbed by mammary alveolar cells for milk fat synthesis; and (b) Mobilized body fat: This includes non-esterified FAs, such as PA, stearic, and oleic acids from adipose tissue [32]. Of the total dietary FAs absorbed in the small intestine, 50%–60% are transferred to milk, whereas 10% are incorporated into milk fat [28]. This high transfer efficiency of dietary FAs likely explains the observed increase in milk fat following PA supplementation in the present study.

Delavaud *et al*. [25] observed that supplementation with hydrogenated PA in goats increased the concentration of C16: 0 in blood plasma, a finding consistent with Lévesque *et al*. [24], who reported a similar rise in C16: 0 in the FA profile of goat milk. This outcome aligns with our study and earlier research in dairy cows by Piantoni *et al*. [26]. The increase in C16: 0 can be attributed to its enhanced bioavailability and preferential incorporation during *de novo* mammary synthesis. During triglyceride formation in milk, C16: 0 is favored at the sn-1 position during acylation [19].

Previous studies on dairy goats [24, 25] and dairy cows [13] also reported a reduction in FA <C16: 0 with PA supplementation. Although the concentrations of medium-chain FAs (C6: 0, C8: 0, C10: 0, and C12: 0) are naturally higher in goat milk than in cow milk due to greater acetate polymerization in the rumen, the reduction of these FAs following PA supplementation was also observed.

This reduction in *de novo* synthesized FAs (<C16: 0) may be explained by the impact of PA supplementation on blood plasma and hormonal responses. PA supplementation increases FA concentrations in blood plasma and stimulates insulin secretion [25]. Elevated insulin activates acetyl-CoA carboxylase in the mammary gland, promoting medium-chain FA *de novo* synthesis. However, PA inhibits the incorporation of FAs <C16 into triglycerides, favoring the esterification of C16: 0 and C16: 1 at the sn-2 and sn-3 positions of glycerol [19]. Our study supports this mechanism, as PA supplementation decreased FA <C16 while increasing the concentration of C16: 0 and C16: 1 in milk fat. These changes reflect the preferential use of C16: 0 for triglyceride synthesis in the mammary gland, which is consistent with the metabolic and biochemical roles of PA in ruminants.

In goats, Delavaud *et al*. [25] observed an increase in FAs >C16 in blood plasma following PA supplementation, whereas Lévesque *et al*. [24] reported a decrease in these FAs in the milk FA profile, similar to the findings of Piantoni *et al*. [26] and Western *et al*. [13] in dairy cows. This decrease in LCFAs (>C16) in milk following PA supplementation can be explained by the role of PA in increasing ceramide supply in the animal’s system, which may contribute to liver dysfunction and metabolic alterations. These alterations can cause FA accumulation in the liver in the form of diacylglycerol, preventing its transport to the mammary gland. Consequently, the synthesis of LCFAs in milk fat is impaired [29].

The lipid metabolism in tissues, including the transfer of FAs between organs, plays a critical role in determining the composition of milk fat in ruminants [19]. Despite the decrease in LCFAs, the balance between saturated and unsaturated FAs is maintained in milk, which is essential for the proper formation of TGs in milk fat globules. This balance influences the fluidity of milk fat, which is crucial for its physical properties and functionality [9]. Thus, while the concentration of FAs >C16 in milk fat may decrease with PA supplementation, the overall composition and functionality of milk fat are preserved due to the physiological regulation of FA balance in milk.

The increase in milk C16: 0 with PA supplementation (Table 3) is consistent with a substrate-driven mechanism: Dietary C16: 0 is absorbed as chylomicron triacylglycerol and non-esterified FA (NEFA), hydrolyzed by lipoprotein lipase, and taken up by mammary epithelium (via CD36/FATP) for direct incorporation into triacylglycerols [24]. In goats, whose milk contains more short-and medium-chain FA (C6: 0–C12: 0) than bovine milk, the mammary gland also relies heavily on *de novo* lipogenesis from acetate and BHBA through ACC/FASN, producing FA up to C16: 0 [25, 27]. A well-described regulatory feature in ruminant mammary tissue is that a greater inflow of preformed LCFA can down-modulate *de novo* synthesis (product feedback on ACC/FASN) [32]. Our concurrent decline in the <C16 fraction with rising PA (Table 3) fits this model: Exogenous C16: 0 supplies the terminal product of the *de novo* pathway and attenuates upstream synthesis, lowering short-chain proportions without reducing milk volume (Table 2, Figures 1 and 2).

The reduction in the >C16 fraction (C18: 0, C18: 1) under PA feeding (Table 3) likely reflects two processes. First, improved energy balance (Table 4) reduces adipose mobilization, decreasing the mammary inflow of endogenous C18-rich NEFA. Second, intracellular TAG assembly may preferentially esterify palmitate at sn-positions, displacing very-LCFA when C16: 0 supply rises. Although mammary SCD1 desaturated C18: 0→C18: 1 (and C16: 0→C16: 1), the observed net fall in >C16 suggests that desaturation/elongation (C16: 0→C18: 0 via ELOVL) did not offset the reduced mobilization-derived C18 flux [29, 31].

In cows, PA commonly elevates milk fat yield and C16: 0 with neutral DMI; bovine milk often shows a larger baseline contribution from preformed C18, and diet-induced MFD can be pronounced with certain unsaturated-fat/fermentability combinations [26, 28]. Goats are generally less prone to classical MFD, maintain a higher *de novo* medium-chain FA baseline, and sustain milk volume across a wider lipid-inclusion range [8]. Therefore, our pattern is caprine-typical: (1) Stable intake and yield (Table 2), (2) selective enrichment of C16: 0 with concurrent reductions in <C16 and >C16 (Table 3), and (3) improved energy balance without required yield gains (Table 4). Weekly protein and lactose trajectories (Figures 1 and 2) support a shift in substrate use rather than a broad stimulation of secretory capacity.

The selective enrichment of C16: 0 (PA) with proportional declines in Σ < C16 and Σ >C16 (Table 3) modestly increases the saturated-fat share of milk fat [24]. Palmitate is a common dietary SFA; higher proportions may slightly raise atherogenic indices relative to MUFA/PUFA-richer profiles, whereas decreases in short-/medium-chain FA (C6: 0–C12: 0) could reduce some rapidly oxidized lipids associated with quick energy provision [1]. The net nutritional relevance depends on the overall diet context and serving size. A moderate PA inclusion (~3% DM) that achieves energetic benefits (Table 4) while avoiding excessive hardening of the profile (Table 3) is preferable if targeting a balanced SFA/MUFA mix [24].

On the other hand, increasing C16: 0 tends to raise the melting point and promote a more crystalline TAG matrix, which can enhance fat firmness/body and influence cheese yield and texture, while potentially reducing spreadability in high-fat products [33]. Conversely, reductions in Σ <C16 may slightly dampen the intensity of short-chain flavor notes (C6: 0–C10: 0) characteristic of some goat cheeses [33]. In our data, milk volume and routine composition (fat, protein, and lactose) were stable across treatments (Table 2, Figures 1 and 2), so techno-functional changes would primarily arise from fat-phase structuring rather than bulk solids [33]. Practically, rations near 3% PA can improve energy status (Table 4) with manageable effects on texture/flavor; processors seeking softer profiles could blend lots across weeks or adjust make parameters (such as ripening temperature) to accommodate the slightly higher C16: 0.

### Improved energy balance without increasing yield

In the present study, the inclusion of PA in the diet of lactating goats resulted in a noticeable increase in the concentration of C16: 0 in milk fat, demonstrating a positive transfer of PA from the diet to the milk. This finding is consistent with previous research showing that dietary PA supplementation increased milk fat content by facilitating the efficient transfer of FAs from the diet into the mammary gland [34]. The increase in C16: 0, a saturated FA, suggests that PA supplementation provided a source of LCFAs that were efficiently incorporated into milk fat, thereby improving the composition of milk fat and potentially benefiting the milk’s nutritional quality.

PA increases dietary net energy density and can lower the heat increment per unit of energy yielded relative to carbohydrate, improving the ratio of ME captured in milk or retained in body tissues [11, 12]. In our goats, PA improved whole-animal energy balance without increasing milk volume (Table 4), indicating that intake at a given DMI met a greater proportion of the daily requirement for maintenance plus lactation (Table 2). Two processes likely contributed to this process: (1) Partitioning, preferential routing of absorbed C16: 0 to mammary TAG formation (a process that is ATP-efficient once substrate supply is adequate) and (2) sparing of mobilization, a better energy status reduces adipose lipolysis, lowering endogenous C18-rich NEFA flux to the udder [8]. The latter aligns with the observed decline in >C16 FA (Table 3) and a shift away from reliance on mobilization-derived LCFA. Together, these mechanisms provide a biologically coherent explanation for improved energy balance at 3% PA, with little additional gain at 6%, suggesting a physiological plateau in substrate handling and partitioning.

One of the significant metabolic changes observed in this study was the reduction in the NEL required to synthesize milk fat. The enhanced transfer of PA to milk fat decreased the energy demand for *de novo* fat synthesis in the mammary gland [19]. This reduction in NEL may have had several beneficial effects on the goats’ energy metabolism. In particular, it allowed the available energy to be redirected to other physiological processes essential to improving goats’ overall health and productivity [35].

For instance, the energy previously allocated to milk fat synthesis could now be redirected to enhance milk production. This is supported by our findings, which show that goats supplemented with PA exhibited increased ECM production, indicating that energy was efficiently utilized to improve overall milk output [8]. In addition, the reduction in NEL requirements could have contributed to a higher milk protein concentration, as energy is often a limiting factor for protein synthesis during lactation. This suggests that the goats had more resources available for milk protein synthesis, resulting in a more nutritionally balanced milk composition.

Moreover, the decrease in the energy requirement for fat synthesis likely contributed to reduced mobilization of body reserves [35]. In lactating animals, body fat is often mobilized to meet the increased energy demands of milk production. By reducing the energy needed for milk fat synthesis, PA supplementation could have helped preserve body reserves, leading to a more stable body condition in the goats and preventing excessive body weight loss during lactation [36]. This preservation of body reserves is important for the long-term health and reproductive success of the animals, as it ensures an adequate energy store for future lactation cycles and maintains overall metabolic balance.

Another important effect of the reduced NEL requirement was the improvement in the goats’ energy balance. Energy balance is a crucial factor in the health and productivity of dairy animals, and a positive energy balance is linked to enhanced fertility, improved immune function, and overall well-being. In our study, PA-fed goats exhibited an improved energy balance, suggesting that the energy not used for milk fat synthesis was redirected toward supporting other vital functions [8]. This improved energy balance could also explain the observed reduction in DMI in PA-supplemented goats, as they were able to meet their energy needs more efficiently, resulting in less dependency on high DMI for the same level of milk production.

Improved energy balance typically attenuates the metabolic strain associated with negative energy status, reducing circulating NEFA and ketone body pressure, thereby supporting hepatic lipid handling and limiting excessive TG deposition [37]. Goats are less prone to classical diet-induced milk fat depression than cows [8] and often tolerate moderate lipid supplementation without intake depression, which is consistent with our stable DMI and yield (Table 2) and the absence of adverse welfare events noted in the methods. A modest increase in post-absorptive LCFA supply can downregulate hepatic *de novo* lipogenesis and favor mitochondrial β-oxidation, while at the mammary gland, the balance between SCD1-mediated desaturation (C18: 0→C18: 1; C16: 0→C16: 1) and TAG assembly determines the ultimate FA profile [38]. In our data, the net reduction in >C16 suggests that any desaturation/elongation (C16: 0→C18: 0) did not offset the decreased mobilization load, consistent with a healthier energy state rather than an inflammatory or lipotoxic shift [39]. From a practical perspective, this suggests that moderate PA (≈3% DM) can enhance ME and stabilize metabolic status without compromising intake, whereas higher inclusion (6%) provides limited incremental benefit for EB and may unnecessarily harden the FA profile.

The comparative responses at 3% versus 6% PA indicate that the mammary and whole-animal systems likely reached an effective substrate sufficiency of approximately 3%, beyond which additional C16: 0 offered diminishing returns. For nutrition programs, these favors formulating around lower inclusion rates to capture energy-efficiency gains (Table 4) and the desired FA shift (Table 3), while preserving ration flexibility and minimizing potential trade-offs in product quality [33].

The improvement in energy balance with PA (Table 4) is relevant beyond the immediate milk output, as energetic status is a primary driver of body condition trajectories in mid-lactation goats [8]. In practice, narrowing the energy deficit at a given DMI should reduce reliance on adipose mobilization, limit excessive loss of body reserves, and facilitate stabilization or gradual recovery of body condition where forage quality fluctuates [1]. Better body condition is associated with a lower incidence of metabolic strain (excessive NEFA/ketone pressure) and may favor subsequent reproductive performance by supporting ovarian cyclicity and conception in the weeks following the study window [40]. Although our trial did not collect body-condition scores or reproductive endpoints, the observed EB gain at ~3% PA indicates a biologically plausible pathway for carry-over benefits: Improved partitioning and reduced mobilization can translate into healthier post-trial condition, with potential positive impacts on return to estrus and pregnancy rates in herd settings that routinely face seasonal energy shortfalls [40].

When implementing PA, we recommend pairing ration changes with routine body condition scoring (biweekly) and simple metabolic sentinels (milk fat/protein ratio, weight trends). If the body condition is stable or improving while the milk volume remains steady (Table 2), the intervention is likely to achieve its intended energetic effect. Should the condition fail to stabilize, the fiber effectiveness, overall lipid load, and inclusion level (>3%) should be reassessed.

### Limitations

This study was of moderate duration and conducted on a single commercial farm, which may restrict the extrapolation of the findings to other management systems, breeds, or forage conditions. The absence of blood metabolite measurements, such as NEFA, BHBA, glucose, and urea-N, limits direct interpretation of the systemic metabolic status of the goats. Furthermore, reproductive and additional physiological indicators, including post-trial body condition dynamics, inflammatory biomarkers, and rumen fermentation parameters, were not assessed. As a result, potential carry-over effects of PA supplementation on fertility, long-term metabolic health, and overall productive longevity could not be evaluated within the scope of this study.

## CONCLUSION

The inclusion of PA in the diet of mid-lactation goats improved milk fat synthesis and overall energy utilization efficiency without affecting DM intake, body weight, or milk yield. Goats supplemented with 3% and 6% PA exhibited significantly higher milk fat concentration and yield, as well as increased ECM production, compared with the control group. The milk FA profile shifted markedly with PA supplementation, showing higher proportions of C16: 0 and C16: 1, and a corresponding reduction in *de novo* (<C16) and long-chain (>C16) FAs, reflecting the efficient transfer of dietary PA to milk fat. Energy balance improved across all treatments, particularly in goats receiving 3% PA, indicating better nutrient partitioning and reduced reliance on body fat mobilization.

A major strength of this study lies in its controlled experimental design and detailed characterization of milk composition and energy balance under realistic field conditions. However, its single-site design and lack of data on blood metabolites or reproduction limit broader extrapolation and mechanistic interpretation.

Future studies should incorporate metabolic biomarkers, rumen fermentation parameters, and post-lactation reproductive outcomes to elucidate systemic effects and long-term impacts of PA supplementation.

In conclusion, moderate dietary PA supplementation (3% DM) represents a practical and biologically efficient nutritional intervention to improve milk fat yield and energy balance in lactating goats, supporting both animal performance and production sustainability under variable forage systems.

## DATA AVAILABILITY

All the generated data are included in the manuscript.

## AUTHORS’ CONTRIBUTIONS

JNAT and LDGR: Designed and conducted the study and drafted and edited the manuscript. JERB, YBM, and JAMJ: Coordinated and guided the research. ARG and MRM: Coordinated the statistical analysis. MMCG: Coordinated the laboratory analyses. All authors have read and approved the final manuscript.

## References

[1] Goetsch A.L, Zeng S.S, Gipson T.A (2011). Factors affecting goat milk production and quality. Small Rumin. Res.

[2] Zucali M, Lovarelli D, Celozzi S, Bacenetti J, Sandrucci A, Bava L (2020). Management options to reduce the environmental impact of dairy goat milk production. Livest. Sci.

[3] Torres-Hernández G, Maldonado-Jáquez J.A, Granados-Rivera L.D, Wurzinger M, Cruz-Tamayo A.A (2022). Creole goats in Latin America and the Caribbean:A priceless resource to ensure the well-being of rural communities. Int. J. Sustain. Agric. Res.

[4] Granados-Rivera L.D, Maldonado-Jáquez J.A, Domínguez-Martínez P.A, Salinas-Chavira J, Bautista-Martínez Y (2022). Effect of the feeding system (grazing vs. zero grazing) on the production, composition, and fatty acid profile in milk of Creole goats in northern Mexico. Emir. J. Food Agric.

[5] Dos Santos W.M, Guimarães-Gomes A.C, Caldas Nobre M.S, Souza Pereira A.M, Dos Santos Pereira E.V, Olbrich dos Santos K.M, Rolim-Florentino L, Alonso Buriti F.C (2023). Goat milk as a natural source of bioactive compounds and strategies to enhance the amount of these beneficial components. Int. Dairy J.

[6] Zhang K, Zhang L, Zhou R, Zhong J, Xie K, Hou Y, Zhou P (2022). Cow's milk αS1-casein is more sensitizing than goat's milk αS1-casein in a mouse model. Food Funct.

[7] Song N, Chen Y, Luo J, Huang L, Tian H, Li C, Loor J.J (2020). Negative regulation of αS1-casein (CSN1S1) improves β-casein content and reduces allergy potential in goat milk. J. Dairy Sci.

[8] Granados?Rivera L.D, Hernández?Mendo O, Maldonado?Jáquez J.A (2020). Energy balance in lactating goats:Response to mixture of conjugated linoleic acid. Anim. Sci. J.

[9] Loften J.R, Linn J.G, Drackley J.K, Jenkins T.C, Soderholm C.G, Kertz A.F (2014). Invited review:Palmitic and stearic acid metabolism in lactating dairy cows. J. Dairy Sci.

[10] Vyas D, Moallem U, Teter B.B, Fardin-Kia A.R.K, Erdman R.A (2013). Milk fat responses to butterfat infusion during conjugated linoleic acid-induced milk fat depression in lactating dairy cows. J. Dairy Sci.

[11] De Souza J, Lock A.L (2018). Long-term palmitic acid supplementation interacts with parity in lactating dairy cows:Production responses, nutrient digestibility, and energy partitioning. J. Dairy Sci.

[12] Rico J.E, De Souza J, Allen M.S, Lock A.L (2017). Nutrient digestibility and milk production responses to increasing levels of palmitic acid supplementation vary in cows receiving diets with or without whole cottonseed. J.Anim. Sci.

[13] Western M.M, De Souza J, Lock A.L (2020). Effects of commercially available palmitic and stearic acid supplements on nutrient digestibility and production responses of lactating dairy cows. J. Dairy Sci.

[14] Bautista-Martínez Y, Maldonado-Jáquez J, Orzuna-Orzuna J, Arenas-Báez P, Granados-Rivera L ((2025)). Palmitic acid in the diet of dairy goats and its effect on physicochemical characteristics and fatty acid profile of goat kid meat. Anim. Sci. J.

[15] NRC (2007). Nutrient requirements of small ruminants:Sheep, Goats, Cervids, and New World Camelids.

[16] AOAC Association of Official Agricultural Chemists (2005). Official Methods of the Association of the Agricultural Chemists.

[17] Van Soest P.J, Roberson J.B, Lewis B.A (1991). Methods for dietary fiber, neutral detergent fiber, and nonstarch polysaccharides in relation to animal nutrition. J. Dairy Sci.

[18] Feng S.A, Lock L.A, Garnsworthy P.C (2004). A rapid lipid separation method for determining fatty acid composition of milk. J. Dairy Sci.

[19] Granados-Rivera L.D, Hernández-Mendo O, González-Muñoz S.S, Burgueño-Ferreira J.A, Mendoza-Martínez G.D, Arriaga-Jordán C.M (2017). Effect of palmitic acid on the mitigation of milk fat depression syndrome caused by trans-10, cis-12-conjugated linoleic acid in grazing dairy cows. Arch. Anim. Nutr.

[20] NRC (2001). Nutritional Requirements of Dairy Cattle.

[21] Weiss W.P (1993). Method estimates available energy value for ruminants. Feedstuffs.

[22] NRC (1989). Nutrient Requirements of Dairy Cattle.

[23] Nsahlai I, Goetsch V.A.L, Luo J, Johnson Z.B, Moore J.E, Sahlu T, Owens F.N (2004). Energy requirements for lactation of goats. Small Rumin. Res.

[24] Lévesque J, Dion S, Rico D.E, Brassard M. È, Gervais R, Chouinard P.Y (2022). Milk yield and composition in dairy goats fed extruded flaxseed or a high-palmitic acid fat supplement. J. Dairy Res.

[25] Delavaud C, Fougère H, Bertrand-Michel J, Bernard L (2022). Milk fat depression and plasma lipids in dairy cows and goats. Animal.

[26] Piantoni P, Lock A.L, Allen M.S (2013). Palmitic acid increased yields of milk and milk fat and nutrient digestibility across production levels of lactating cows. J. Dairy Sci.

[27] Fougère H, Delavaud C, Bernard L (2018). Diets supplemented with starch and corn oil, marine algae, or hydrogenated palm oil differentially modulate milk fat secretion and composition in cows and goats:A comparative study. J. Dairy Sci.

[28] Lock A.L, Preseault C.L, Rico J.E, DeLand K.E, Allen M.S (2013). Feeding a C16:0-enriched fat supplement increased the yield of milk fat and improved conversion of feed to milk. J. Dairy Sci.

[29] McFadden J.W, Rico J.E (2019). Invited review:Sphingolipid biology in the dairy cow:The emerging role of ceramide. J. Dairy Sci.

[30] Palmquist D.L, Jenkins T.C (2017). A 100-year review:Fat feeding of dairy cows. J. Dairy Sci.

[31] Harvatine K.J, Boisclair Y.R, Bauman D.E (2009). Recent advances in the regulation of milk fat synthesis. Animal.

[32] Chilliard Y, Ferlay A (2004). Dietary lipids and forages interactions on cow and goat milk fatty acid composition and sensory properties. Reprod. Nutr. Dev.

[33] Granados-Rivera L.D, Hernández-Mendo O, Burgueño-Ferreira J.A, González-Muñoz S.S, Mendoza-Martínez G.D, Mora-Flores J.S, Arriaga-Jordán C.M (2018). Mexican tropical cream cheese yield using low-fat milk induced by trans-10, cis-12 conjugated linoleic acid:Effect of palmitic acid. CYTA J Food.

[34] Bauman D.E, Harvatine K.J, Lock A.L (2011). Nutrigenomics, rumen-derived bioactive fatty acids, and the regulation of milk fat synthesis. Annu. Rev. Nutr.

[35] Lourencon R.V, Patra A.K, Ribeiro L.P.S, Puchala R, Wang W, Gipson T.A, Goetsch A.L (2024). Effects of the level and source of dietary physically effective fiber on feed intake, nutrient utilization, heat energy, ruminal fermentation, and milk production by Alpine goats. Anim Nutr.

[36] Oliveira T.S, Rodrigues M.T (2021). Quantification of mobilization of body nitrogen and protein requirements of dairy goats in early lactation. Livest. Sci.

[37] Ghavipanje N, Fathi Nasri M.H, Farhangfar S.H, Ghiasi S.E, Vargas-Bello-Pérez E (2021). Regulation of nutritional metabolism in transition dairy goats:Energy balance, liver activity, and insulin resistance in response to berberine supplementation. Animals (Basel).

[38] Guo Y, Wei Z, Zhang Y, Cao J (2024). Research progress on the mechanism of milk fat synthesis in cows and the effect of conjugated linoleic acid on milk fat metabolism and its underlying mechanism:A review. Animals.

[39] Yakan A, Özkan H, Çamdeviren B, Kaya U, Karaaslan I, Dalkiran S (2021). Expression patterns of major genes in fatty acid synthesis, inflammation, oxidative stress pathways from colostrum to milk in Damascus goats. Sci. Rep.

[40] Kaewlamun W, Grimard B, Duvaux-Ponter C, Ponter A.A (2020). Kick-starting ovarian cyclicity by using dietary glucogenic precursors in post-partum dairy cows:A review. Int. J. Vet. Sci. Med.

